# Removing the Graduate Record Examination as an Admissions Requirement Does Not Impact Student Success

**DOI:** 10.3389/phrs.2022.1605023

**Published:** 2022-09-26

**Authors:** Lisa M. Sullivan, Amanda A. Velez, Nikki Longe, Ann Marie Larese, Sandro Galea

**Affiliations:** School of Public Health, Boston University, Boston, MA, United States

**Keywords:** workforce development, diversity, GRE, admissions requirements, MPH

## Abstract

**Background:** The predictive validity of components of the Graduate Record Examination (GRE) on student success is inconsistent, and the test itself has been shown to be a barrier for prospective students historically underrepresented in graduate programs.

**Policy Options and Recommendations:** We analyzed three admissions cycles for the Master of Public Health degree at the Boston University School of Public Health before (2016, 2017, 2018) and after (2019, 2020, 2021) eliminating the GRE for diversity and quality of applications, student success, and employment outcomes. We observed increases in diversity (e.g., 7.1% and 7.0% self-identified as African American/Black and Hispanic before eliminating the GRE as compared to 8.5% and 8.2% after), but no loss of quality, as measured by undergraduate grade point averages (GPAs) (e.g., median undergraduate GPA before and after eliminating the GRE of 3.4). We also saw no difference in performance in required core courses (e.g., more than 93.5% of students earned passing grades in required courses before and 94.5% after eliminating the GRE) and graduate employment (i.e., 93.1% employed within 6 months of graduation before and 93.8% after eliminating the GRE). We recommend removing the GRE as an admission requirement for the MPH as a step toward diversifying the public health workforce. This change alone is necessary but insufficient. We also need to develop support programs, tailored specifically to the needs of our future students, to ensure their success.

**Conclusion:** Eliminating the GRE as an admissions requirement for prospective students does not result in loss of student quality or worse program performance.

## Background

The Graduate Record Examination (GRE) General Test assesses verbal, quantitative, critical thinking, and writing skills and has been required in applications for most graduate programs in the United States, including master’s and doctoral programs in public health. Over the past decade, numerous programs changed their admissions requirements, with some dropping the GRE entirely, and others making it optional [1]. There were two principal motivations for this change: preliminary evidence that the GRE may not correlate with success in graduate programs, and that the GRE was a barrier to admission for students who have been historically underrepresented in graduate programs. The COVID-19 pandemic added an additional motivation to drop GRE as an admissions requirement as many prospective students could not access testing centers, hindering their ability to complete their applications.

Boston University School of Public Health (BUSPH) aims to recruit, retain and support the success of a diverse student body in all of our programs, seeing diversity and inclusion as essential to fulfilling our mission as an academic public health institution; a mission firmly rooted in social justice. We therefore aim to remove any barriers that interfere with recruitment, retention and success of diverse students. One such barrier was the GRE, which prior to 2019, was a required component for admission to all of our graduate degree programs (i.e., Master of Public Health (MPH), Master of Science (MS), PhD and DrPH). Beginning in fall of 2019, we eliminated the GRE requirement for admission based on three concerns.

First, the GRE is financially burdensome. The cost of the general examination is over $200, there are additional fees to send scores to specific programs [2], and GRE tutoring and preparatory courses can cost in excess of thousands of dollars.

Second, the GRE has been shown to disadvantage women and minority students, particularly in science, technology, engineering and mathematics (STEM) fields [3]. Standardized tests in general, including the GRE, have been shown to be systematically biased with test scores associated with socioeconomic status, race and gender [4]. The Educational Testing Service (ETS), the organization that administers the exam, reports that women score on average 80 points lower than men, African Americans some 200 points lower than whites [5].

Third, the correlation between GRE scores and academic success in graduate school is weak [6]. A meta-analysis of over 1700 studies reported weak, even negative, correlations between GRE scores and measures of success in graduate programs such as degree attainment, time to completion, and research publications and presentations [7]. Internal analysis of data from BU MPH students showed no significant differences in mean GRE component scores between first year students who failed to achieve an overall 3.0 GPA and those who did not (data not shown).

Therefore, in 2019 we eliminated the GRE for a pilot period of 3 years. During that time, we evaluated application trends with respect to diversity and quality, tracked student success in terms of academic performance in required core courses and in graduate outcomes as reflected in employment statistics. Here we present data on student diversity and success in our MPH program before and after eliminating the GRE requirement.

## Evidence

This analysis considers data on all MPH program applications for fall admission cycles from 2016 through 2021. This represents three cycles before we eliminated the GRE (2016, 2017, 2018) and three after (2019, 2020, 2021).

Data were merged from three sources, the Schools of Public Health Application Service (SOPHAS), the university information system, and alumni surveys. Demographic data, including sex, date of birth, race, ethnicity, and US citizenship were self-reported on SOPHAS applications. We added two additional, custom questions to our application to assess if applicants were first in their families to complete an undergraduate or a graduate degree. Applicants also enter all courses completed, credits and grades earned. SOPHAS verifies these data and calculates standardized, semester-based, grade point averages (GPAs) by assigning numeric grades to letter grades, summing these and weighting by course credits. Each course is then categorized into a subject area (e.g., biology/chemistry/physics/life science, business, health science, math/statistics, other, public health, social/behavioral) and subject-specific GPAs are computed using the same approach [8]. Grades in required courses for the MPH were extracted from our university information system and merged with SOPHAS data by unique student identification numbers assigned at application. Employment status within 6 months of graduation is gathered by alumni surveys which are sent *via* e-mail and LinkedIn.

For each cycle, we summarized demographic characteristics of applicants (see Table 1). We tested for significant differences in categorical and quantitative demographic characteristics before and after eliminating the GRE using chi-square tests and analysis of variance, respectively. Following the elimination of the GRE, we observed a significant difference (*p* < 0.001) in the numbers of applicants who self-identified as African American/Black (8.5% versus 7.1%), Asian (15.1% versus 15.0%), and Hispanic (8.2% versus 7.0%), and significantly higher percentages of first-generation graduate school applicants (38.8% versus 36.5%, *p* = 0.012). We also received applications from students who completed undergraduate degrees at institutions not previously represented (data not shown). Quality of applications, based on undergraduate GPA overall and GPA in math/statistics, was not different before (median 3.4 overall and 3.2 in math/statistics) and after (median 3.4 overall and 3.2 in math/statistics) eliminating the GRE (*p* = 0.375 and *p* = 0.510, respectively).

**TABLE 1:** Characteristics of applicants by admission cycle (Boston, Massachusetts. 2022).

	GRE required for admission				GRE not required for admission				
Application cycle	2016	2017	2018	2016–2018	2019	2020	2021	2019–2021	p
Applications to BUSPH MPH	1583	2074	1934	5591	1490	2023	2506	6019	
Female, %	79.6%	80.2%	80.7%	80.2%	78.7%	82.5%	82.3%	81.5%	<0.001
Age, years									
Mean (min-max)	24.4 (19–51)	24.5 (20–65)	24.4 (19–69)	24.4 (19–69)	24.4 (19–69)	24.5 (19–54)	24.3 (19–56)	24.4 (19–69)	0.488
Race/Ethnicity									
African American/Black	7.5%	6.9%	7.0%	7.1%	7.6%	8.9%	8.6%	8.5%	<0.001
Asian	14.7%	14.7%	15.5%	15.0%	16.1%	14.1%	15.3%	15.1%	
Hispanic	6.7%	7.1%	7.3%	7.0%	8.2%	6.8%	9.2%	8.2%	
White	40.9%	39.7%	39.8%	40.1%	38.7%	36.8%	39.2%	38.3%	
Two or more races	2.2%	3.2%	2.7%	2.7%	3.0%	3.3%	2.9%	3.0%	
Non-resident Alien	21.5%	22.4%	22.6%	22.2%	24.3%	27.4%	22.5%	24.5%	
US citizenship, %	75.3%	75.6%	75.2%	75.4%	72.6%	71.2%	75.6%	73.4%	<0.001
1st Gen Undergraduate, %	13.5%	14.7%	16.2%	14.9%	14.4%	15.5%	15.6%	15.3%	0.593
1st Generation Graduate, %	35.5%	36.9%	37.0%	36.5%	38.0%	40.0%	38.5%	38.8%	0.012
Undergraduate GPA									
Median (Q1-Q3)	3.5 (3.2–3.8)	3.5 (3.2–3.7)	3.5 (3.2–3.8)	3.4 (3.2–3.8)	3.5 (3.1–3.8)	3.5 (3.2–3.8)	3.5 (3.2–3.8)	3.4 (3.2–3.8)	0.375
Undergraduate Math GPA									
Median (Q1-Q3)	3.2 (2.7–3.7)	3.2 (2.7–3.7)	3.2 (2.7–3.7)	3.2 (2.8–3.7)	3.2 (2.6–3.7)	3.3 (2.6–3.7)	3.3 (2.7–3.8)	3.2 (2.8–3.8)	0.510

To assess student success, we evaluated student performance in four integrated core courses, required of all MPH students, regardless of their choice of specialization. Students must earn a minimum grade of B- in each core course in order to progress in the program. Students who do not earn the minimum grade must retake the course. The required courses are: Quantitative methods for public health; Leadership and management in public health; Health systems, law, and policy; and Individual, community and population health. In Table 2 we summarize student performance in each core course, specifically, the percentages of students who passed each course, failed to earn the minimum required grade of B-, and who withdrew for each application cycle (see Table 2). Withdrawals occur for a number of reasons. In some instances, students experience an unforeseen life event and need to withdraw, in others, students are struggling and advised to withdraw if they are at risk for failing the course and might need time to focus on other courses. Unfortunately, data are not available to distinguish these different scenarios. We tested for significant differences in student performance before and after eliminating the GRE using Fisher’s exact test.

**TABLE 2:** Student success in integrated core courses (Boston, Massachusetts. 2022).

	GRE required for admission				GRE not required for admission				
Application cycle	2016	2017	2018	2016–2018	2019	2020	2021	2019–2021	p
New enrollments	278	398	317	993	202	371	415	998	
Quantitative methods for public health									
% Passing	93.9%	97.7%	94.2%	95.6%	95.5%	93.2%	94.8%	94.5%	0.032
% Withdrawing	1.8%	0%	1.6%	1.0%	1.5%	1.6%	4.2%	2.5%	
% Failing to earn minimum grade of B-	4.3%	2.3%	4.1%	3.4%	3.0%	5.1%	1.0%	2.9%	
Leadership and management for public health									
% Passing	98.9%	99.0%	100.0%	99.3%	99.0%	99.2%	99.7%	99.4%	0.331
% Withdrawing	1.1%	0.3%	0%	0.4%	0%	0%	0.3%	0.1%	
% Failing to earn minimum grade of B-	0%	0.8%	0%	0.3%	1.0%	0.8%	0%	0.5%	
Health systems, law, and policy									
% Passing	86.0%	94.9%	98.4%	93.5%	96.5%	98.6%	98.3%	98.2%	<0.001
% Withdrawing	2.5%	1.3%	0.3%	1.3%	0.5%	0.3%	1.1%	0.6%	
% Failing to earn minimum grade of B-	11.5%	3.8%	1.3%	5.2%	3.0%	1.1%	0.6%	1.2%	
Individual, community, and population health									
% Passing	99.3%	99.2%	99.7%	99.4%	99.5%	98.9%	99.2%	99.2%	0.610
% Withdrawing	0.7%	0.3%	0.3%	0.4%	0.5%	0.3%	0.3%	0.3%	
% Failing to earn minimum grade of B-	0%	0.5%	0%	0.2%	0%	0.8%	0.5%	0.5%	

Student success remained strong after eliminating the GRE as an admissions requirement, as evidenced by performance in required core courses for the MPH. Percentages of students passing core courses ranged from 95.6% to 99.4% before eliminating the GRE and from 94.5% to 99.5% after eliminating the GRE. We did observe statistically significant differences in performance in two of four core courses, with fewer students failing after eliminating the GRE.

**FIGURE 1:**
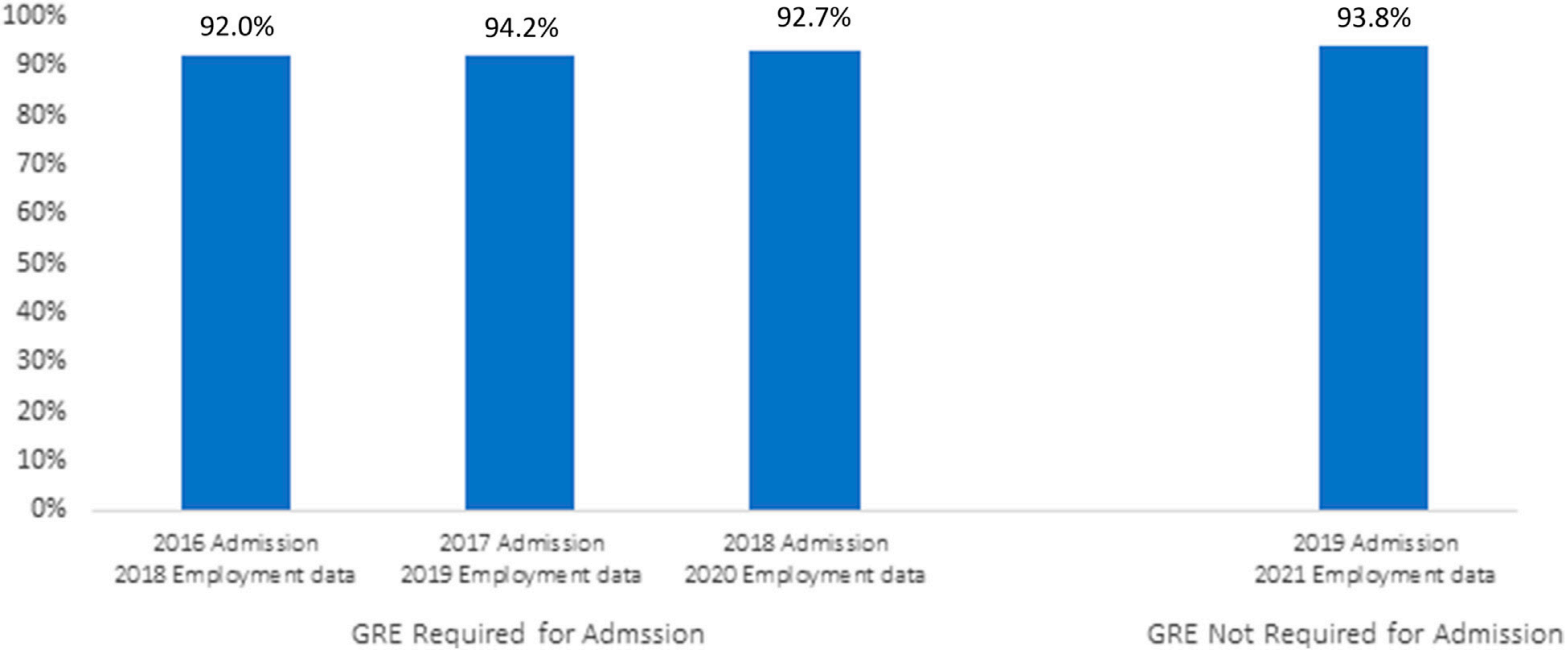
Student employment within 6 months of graduation (Boston, Massachusetts. 2022).

As a graduate professional program, BUSPH aims to prepare students for the public health workforce. We annually gather and analyze employment data by tracking student employment within 6 months of graduation. Our modal student completes the MPH program in 2 years. Thus, for each application cycle, we linked the employment data 2 years later. For example, for the 2016 admission cycle, we present the 2018 employment data. In Figure 1, we summarize employment data before and after the elimination of the GRE. For the 3 years prior to eliminating the GRE, we observed 92.0%, 94.2% and 92.7% of students employed within 6 months of graduation. For the first cohort where the GRE was not required (2019), we observed 93.8% employed within 6 months of graduation. There was no statistically significant difference in employment before versus after eliminating the GRE (*p* = 0.831). We are continuing to monitor graduate employment data for the 2020 and 2021 admissions cohorts and will report those data when they are available.

## Policy Options and Recommendations

The Association of Schools and Programs of Public Health (ASPPH) reported that in 2021, 59% of schools and programs did not require the GRE in MPH applications, up from 16% the year prior [9]. While we initially wanted to explore the impact of a temporary waiver of the GRE, we suggest that the evidence presented here should suggest the elimination of the GRE permanently as a condition for graduate school admission. The GRE is a potential barrier to the recruitment of a more diverse student body and our data suggest that we can identify applicants who will be successful academically and professionally without the GRE.

There are a number of contextual factors that may have affected our findings. First, the impact of the COVID-19 pandemic on all aspects of life, personal and professional, cannot be understated. Students all over the country reported substantial burden of stress during the pandemic, as courses shifted from being offered in-person, to fully remotely, and then in hybrid modality with some attending in-person and some remotely. Second, the school has increased its scholarship aid over time to meet students’ growing financial needs. Even with the increase, some of our programs remain out of reach for many, suggesting that we may not be evaluating the utility of the GRE for a full spectrum of students who may otherwise apply to a school if cost was not a consideration. Third, as an institution, we have several key programs in place that are important to note. These include, but are not limited to, our core course tutoring program, peer coach writing program, and our new mentoring program for first generation students. All programs are free to students and managed through the BUSPH Office of Graduate Student Life. The core course tutoring program is a peer tutoring program for any MPH student at risk of failing to meet the required minimum grade in a core course. Interested students are matched with a peer tutor who works with them one-on-one, to provide assistance with course content and assessments. Tutors are trained and supported by the school. The peer coach writing program offers student-directed writing appointments to students enrolled in any SPH course, including the core courses. Students can work with a peer writing coach to brainstorm ideas or angles to approach specific topics, receive feedback on outlines and drafts, and to navigate assignment rubrics. Peer writing coaches are trained and supported by the school. In fall of 2021, the school launched a mentoring program for first generation students to help them navigate the often complex system of higher education. Students self-identify for the program and are matched with faculty and staff mentors who themselves experienced graduate school as a first generation student. Faculty and staff are provided training and the school offers resources to mentors to support their meeting and working with mentees. It is possible that programs without these supports may find that some students, who might have previously not be admitted due to poor GRE scores, may struggle to succeed in graduate school.

## Conclusion

BUSPH eliminated the GRE as an admissions requirement in 2019, motivated primarily by our aspirations to ensure equitable admission opportunity for all. We examined three admissions cycles before and three admissions cycles after eliminating the GRE for diversity and quality of applications, student academic success in required core courses, and employment within 6 months of graduation. Diversity of applicants increased slightly, quality remained high, students performed well in required core courses, and graduate employment data remained strong. Thus, we will not require the GRE for future admissions cycles. There is more work to do to ensure that we recruit, retain, and support a diverse student body. For example, we will continue to enhance programs to better support students historically underrepresented in graduate programs and first generation students, financially, academically, personally and professionally. The future of the public health workforce will be directly affected by the decisions academic institutions make to maintain or reduce barriers to access advanced public health degree programs and we support the removal of the GRE as an admission requirement as a way to eliminate one such barrier.

## References

[1] Langlin K. A Wave of Graduate Programs Drops the GRE Application Requirement. Science (2019). Available at: https://www.science.org/content/article/wave-graduate-programs-drop-gre-application-requirement (Accessed July 26, 2022).

[2] Educational Testing Service. Fees for GRE Tests and Related Services (2021). Available at: https://www.ets.org/gre/revised_general/register/fees (Accessed April 5, 2022).

[3] Miller C Stassun K. A Test that Fails. Nature (2014) 510:303–4. 10.1038/nj7504-303a

[4] Rosales J Walker T. The Racist Beginnings of Standardized Testing. Washington, DC: National Education Association (2021). Available at: https://www.nea.org/advocating-for-change/new-from-nea/racist-beginnings-standardized-testing (Accessed April 5, 2022).

[5] Tyson C. STEM Graduate Programs Place Too Much Emphasis on GRE Scores, Physicists Say. Washington, DC: Inside Higher (2014). Available at: https://www.insidehighered.com/news/2014/06/16/stem-graduate-programs-place-too-much-emphasis-gre-scores-physicists-say (Accessed April 5, 2022).

[6] Ethridge B. Correlation Etween the GRE and Graduate School Performance (2018). Available at: https://www.dominatethegre.com/2018/07/gre-correlation-to-graduate-school-performance (Accessed April 5, 2022).

[7] Kuncel NR Hezlett SA Ones DS. A Comprehensive Meta-Analysis of the Predictive Validity of the Graduate Record Examinations: Implications for Graduate Student Selection and Performance. Psychol Bull (2001) 127(1):162–81. 10.1037/0033-2909.127.1.162 11271753

[8] Schools of Public Health Application Service. Calculating Your SOPHAS GPAs (2017). Available at: https://help.liaisonedu.com/SOPHAS_Applicant_Help_Center/Submitting_and_Monitoring_Your_SOPHAS_Application/Verification_and_GPA_Calculations_for_SOPHAS/3_Calculating_Your_SOPHAS_GPAs#Overview (Accessed July 26, 2022).

[9] Association of Schools and Programs of Public Health. GRE Scores and Public Health Admissions (2021). Available at: https://s3.amazonaws.com/ASPPH_Media_Files/Docs/ASPPH_GRE+Position+Paper.pdf (Accessed April 5, 2022).

